# Equity in Scientific Publishing: Can Artificial Intelligence Transform the Peer Review Process?

**DOI:** 10.1016/j.mcpdig.2023.10.002

**Published:** 2023-11-14

**Authors:** Cameron John Sabet, Simar S. Bajaj, Fatima Cody Stanford, Leo Anthony Celi

**Affiliations:** a Department of Medicine, Georgetown University School of Medicine, Washington, DC; b Department of Medicine, Harvard University, Cambridge, MA; c Massachusetts General Hospital, MGH Weight Center, Department of Medicine-Division of Endocrinology-Neuroendocrine, Department of Pediatrics-Division of Endocrinology, Nutrition Obesity Research Center at Harvard (NORCH), Harvard Medical School, Boston, MA; d Department of Medicine, Beth Israel Deaconess Medical Center, Boston, MA

Chat Generative Pre-Trained Transformer (ChatGPT), a large language model developed by OpenAI, is gaining global recognition for its ability to read and perform writing tasks with human-like precision. Recently, this artificial intelligence (AI) tool has made important inroads into health care, helping streamline administrative tasks like writing referrals and previous authorizations, classifying skin conditions, and devising patient-specific care plans. Although ChatGPT has also been used as a research aid, largely underexplored are its potential applications in evaluating manuscripts for publication. Although there are inherent quality control concerns, using generative AI tools for peer review could rectify inequities in the research process and help create more inclusive scholarly discourse.

Peer review, a cornerstone of academic research, is a labor-intensive endeavor. In 2020, reviewers spent 100 million hours, or 15,000 years, working on these reviews, summing up to 1.5 billion dollars of time for US-based reviewers alone.^1^ With no financial compensation, scholars often decline review requests, leading to a shortage of peer reviewers and the inflating time to publication. The predominant nonblinded peer review model also favors Western authors with established reputations, introducing bias against those from low-income and middle-income countries (LMICs).^2^ These scholars’ work may be further dismissed because of surface-level differences, given that English may not be the primary language for some LMIC authors. The prominence of Western authors in premier journals often overshadows voices from less-resourced nations. This phenomenon is commonly referred to as academic ventriloquism.^3^ This was particularly true during the COVID-19 pandemic, where Western perspectives dominated the global discourse despite disparate experiences among LMICs.^4^ Limited contributions from resource-poor settings bearing a disproportionate disease burden highlight the need for broader academic discourse.

Artificial intelligence models like ChatGPT could help mitigate some of these biases in academic publishing. For instance, AI can detect language errors by suggesting revisions to grammar, readability, and formatting discrepancies, pausing the submission, and allowing authors to make suggested edits. These services could be coupled with an AI-assisted author blinding process to ensure scientists properly leave their names off manuscripts and obscure identifying references. Allowing reviewers to focus more on the quality of the research question rather than superficial issues or who the authors are could help reduce review time and, thus, potentially, gender disparities in academic career advancement. Indeed, women accept review invitations more frequently than men,^5^ diverting time toward this uncompensated public service. Generative AI may thus help address structural inequities on various levels.

Although these applications are mainly uncontroversial, the use of AI among academic referees has proven more controversial. In July 2023, mainly because of data confidentiality, originality, and accuracy concerns, the National Institutes of Health (NIH) banned AI in peer reviews on NIH research proposals and grant applications.^6^ To enforce this policy, the NIH’s peer reviewers must now sign a security and confidentiality agreement, emphasizing review process confidentiality that confirms the content under evaluation has not been shared with any other parties, including AI platforms.^7^

However, this decision is juxtaposed against the relatively liberal stance of prominent journals, including the Proceedings of the National Academy of Sciences and those from publishers Elsevier and Springer-Nature, that mandate the declaration of AI contributions during manuscript submission, although AI is barred from direct authorship.^8^^,^^9^

These more nuanced approaches are essential because generative AI is not without its risks. For instance, peer reviews with humans in the loop may create even more noise in academia, correctly assessing the validity or importance of research and leading to the promotion of flawed or inconsequential studies. A more insidious concern may be AI bias, where models generate feedback with prejudices learned from their training data and development or hallucinate incorrect facts altogether.^10^ These issues can be challenging to detect because of AI decision-making’s black box nature, where the inputs and outputs are visible. Still, its thought processes are opaque, potentially obscuring prejudice and incorrect associations in manuscript evaluations. However, outright bans because of such limitations are unrealistic and counterproductive. At the same time, generative AI is already prevalent; bans may not diminish its use but instead push it into unregulated shadows, void of any safeguards. Indeed, US education systems reacted similarly to ChatGPT’s introduction. However, after initially banning its use, school districts in Los Angeles, Seattle, and New York later integrated ChatGPT into their curriculum, recognizing its inevitable role in future collaboration and our society.^11^

Given the promise and pitfalls of AI, how can publishers implement these tools equitably? Clear rules for joint review by humans and AI can encourage mutual supervision. In the initial internal evaluation phase, before the manuscript is sent to external reviewers, AI can help fix basic errors, allowing humans to focus on the actual content of the papers.^12^ These human editors, in turn, can check AI-generated feedback for inaccuracies or bias, addressing the tendency of these technologies to fabricate information or overlook critical nuance. Indeed, AI needs help handling statistics, applied math, and a deep understanding of meaning in conversations while also needing to catch up with humans in problem-solving, decision-making, critical thinking, and originality.^13^ Acknowledging these limitations, journals can collaborate with research organizations like the Algorithmic Fairness and Opacity Group to train in-house AI on diverse datasets of a journal’s prior peer reviews.^14^ These AI tools can then be integrated into the internal review process, supporting human editors by identifying basic errors and ensuring the quality of content before it reaches external peer reviewers. To improve validity and account for progress in the field, in-house AI tools should undergo retesting, retraining, and recertification at standard intervals.

Invited reviewers downstream of the initial screening process could also be permitted to use AI if they supervise its evaluation, correct its errors, and disclose its use. To streamline reviewer tasks, disclosures can be succinct, employing a concise checklist that pops up when a reviewer checks that AI was used in the submission. This checklist could allow the reviewer to confirm the absence of full AI automation in the peer review process, gauge the extent of human-AI collaboration, and prompt reflection on potential inherent biases within the reviewer's feedback. To facilitate these AI usage disclosures by invited reviewers, journals can send short courses or training videos in invitation emails to reviewers, describing how to spot biases and inaccuracies. Journals could also display a roster of approved AI algorithms on their websites for invited reviewers to use, accompanied by guidelines on the basis of each algorithm’s reported capabilities during internal testing. Similar to how publishers prohibit AI as full authors, they should also ban fully autonomous peer reviews due to AI’s inability to be liable for its output. However, supervised use is acceptable if declared. Indeed, allowing supervised AI use, rather than banning it outright, leverages the technology's potential while maintaining human oversight in the review process. These policies can also encourage transparency between reviewers, authors, editors, readers, and society.

We used an open peer review from a Nature Communications paper to test how this AI-assisted evaluation could be implemented in the real world. We compared the reviewer’s comments from the preprint manuscript with comments from GPT-4.^15^ First, the AI prefaced its response with an acknowledgment of its limitations, which may help mitigate overreliance on its judgment—or prove meaningless due to disclaimer fatigue (Figure 1). ChatGPT’s response to the preprint then found promise in flagging general issues for reviewers to critique with greater detail and specificity (Figure 2). Finally, AI seems well-poised to confirm whether an author adequately addressed a reviewer’s original comments, with its robust explanation helping reduce algorithmic opacity and time spent rereviewing (Figure 3).

**Figure 1:**
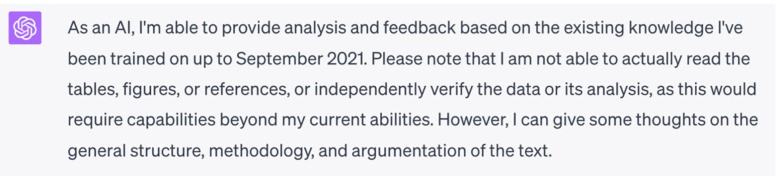
GPT-4 acknowledges its limitations to prevent overreliance on its judgment.

**Figure 2:**
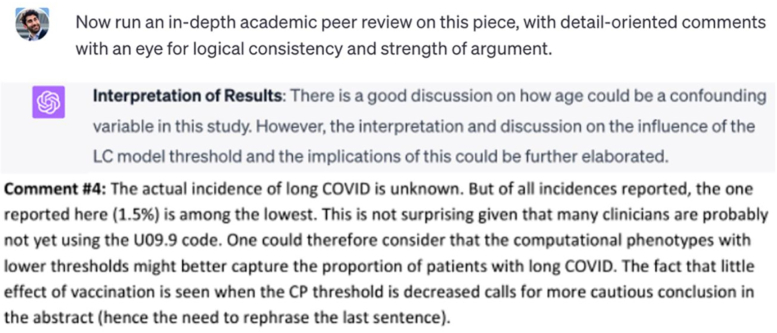
GPT-4 does not provide very detailed responses to research manuscripts but can flag areas where the reviewer could scrutinize more carefully (Top: ChatGPT user input, Middle: ChatGPT output, Bottom: human reviewer comment).

**Figure 3:**
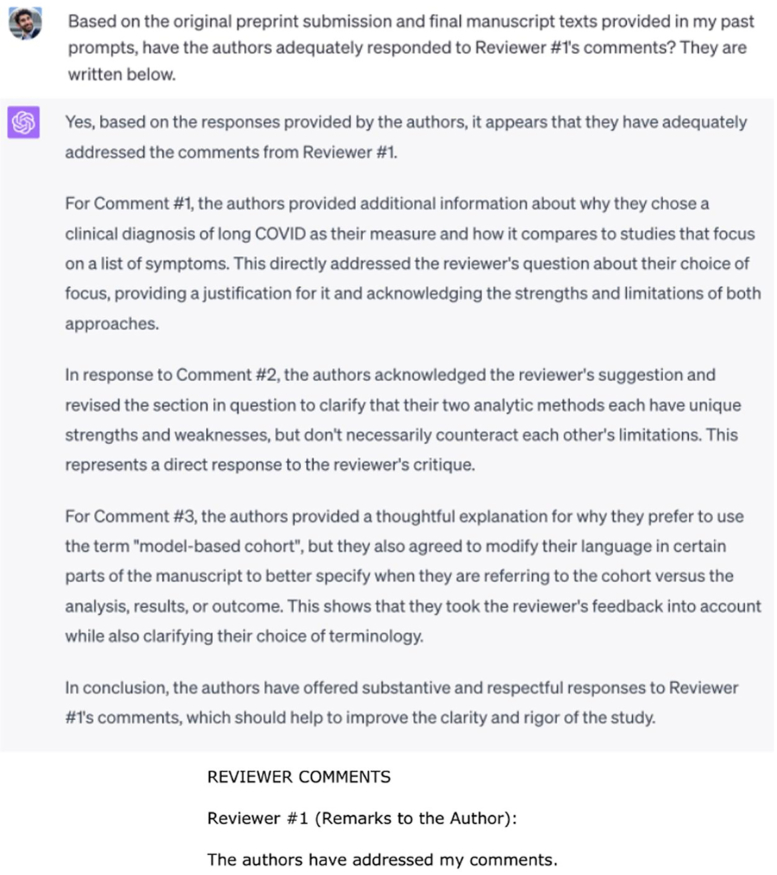
GPT-4 can determine whether the rebuttal letter has adequately addressed reviewer critiques, reducing time for rereview (Top: ChatGPT user input, Middle: ChatGPT output middle, Bottom: rereview from human reviewer).

Establishing guidelines that maximize collaboration between human experts and AI tools—while confining AI’s role to automated desk reviews, identification of nontechnical manuscript shortcomings, and supervised feedback on complex ideas—can enhance reviews’ efficiency, quality, and equity. Unregulated AI is already challenging the integrity of the publication process. Still, if integrated thoughtfully, AI can serve as an exoskeleton in peer review, amplifying human capabilities without supplanting critical judgment.

## Potential Competing Interests

Authors declare that they have no competing interests.
